# Understanding Commitment of Local Food Banks, Faith-Based Organizations, and Schools to Provide Nongovernment Food Programs

**DOI:** 10.1016/j.cdnut.2023.102005

**Published:** 2023-09-25

**Authors:** Eliza M. Fishbein, Edward A. Frongillo, Sharraf Samin, Audrey L. Richards, Christine E. Blake, Ruth P. Saunders, Cheri J. Shapiro

**Affiliations:** 1 Department of Health Promotion, Education, and Behavior, Arnold School of Public Health, University of South Carolina, Columbia, SC, United States; 2 Institute for Families in Society, University of South Carolina, Columbia, SC, United States

**Keywords:** nongovernment food assistance, commitment, values, identity, food bank, faith-based organization, schools, wants, assumptions

## Abstract

**Background:**

Nongovernment food assistance is typically provided to families by faith-based organizations, schools, and food banks. Community organizations appear to be strongly committed to these programs, but little is known about the basis for this commitment.

**Objectives:**

The aim of this study was to examine the values and identities of community organizations to understand the reasons for their commitment to providing nongovernment food assistance.

**Methods:**

Thirty-three in-depth interviews were conducted with 36 leaders at faith-based organizations (19 participants), schools (14 participants), and a local food bank (3 participants) in South Carolina. Observations were made, and informational documents (e.g., flyers and pamphlets) were reviewed. Thematic coding using the constant comparative method was guided by the policy concepts of organizational perspectives, values, and identities.

**Results:**

Nongovernment food programs offered participants volunteering opportunities to become involved with community organizations, which in turn increased financial support for the sustainability of these programs. School participants regarded themselves as a mechanism through which food programs were provided because of their commitment to students and believed they have limited capacity to make proposals to influence the food programs. Seeking to improve the well-being of the community by ending hunger was not the primary value on which organizations focused; instead, it was the process of fulfilling other values (e.g., forming or maintaining relationships within the community), maintaining identity, and appealing to their participants that strengthened their commitment to nongovernment food programs.

**Conclusion:**

Nongovernment programs are meant to be a solution to food insecurity complementary to government programs. Commitment to nongovernment programs fulfills organizational identities, wants, and assumptions, but a consequence of commitment to food programs, derived from fulfilling other values, is that the roots of hunger in a community become obscured and alternative solutions are ignored or rejected.

## Introduction

Food insecurity persists in the United States despite the presence of effective government-supported food programs aimed at reducing food insecurity such as the Special Supplemental Nutrition Program for Women, Infants, Children (WIC), the National School Lunch Program, and the Supplemental Nutrition Assistance Program (SNAP) [1]. According to the USDA’s Economic Research Service, in 2021, 56% of households experiencing food insecurity were enrolled in at least 1 of the 3 major federal food-assistance programs [2]. Many nutrition and food-assistance programs have been implemented in the United States during the last few decades intending to reduce food insecurity and hunger among low-income individuals and households [3], some not funded by the government.

Sustaining nongovernment food programs requires significant support from communities [4]. In the United States, faith-based organizations and schools provide logistical support for local food banks [5]. Across the country, a network of food banks addresses hunger via collaborative efforts with connected food pantries, shelters, and community-based organizations. For instance, Feeding America acquires food from corporate manufacturers, merchants, and produce suppliers on a nationwide scale and offers its member food banks technical support, such as guidance on how to maximize enrollment in various nutrition aid programs [6]. Faith-based organizations, through the work of their ministries and volunteers, provide logistical support, finances, and other resources for solutions they support. Schools provide ready access to families and children for food distribution.

Sustaining nongovernment food programs by these organizations suggests commitment to providing these solutions, although little is known about the basis for this commitment, i.e., “the will to act and to keep acting until the job is done” [7,8]. Commitment is a binding pledge, with the obligation that organizations are dedicated and will push through setbacks and follow through with their commitment until they feel their job is completed. This commitment is often the backbone of an organization, giving its members strength and a common interest through which to direct their efforts [9]. Organizational commitment may be linked by organizations’ orientation to the problem and their organizational perspectives, i.e., their identities, what they want from their commitment, and what they assume about their commitment [[9], [10], [11], [12], [13], [14], [15], [16]]. Organizations cooperate more when they share a commitment, as doing so can help to foster camaraderie, trust, and caring [8,9,13]. In the context of nongovernment food programs, the greater basis there is for commitment, the greater the momentum that can be generated to provide food programs.

Besides solving a community need, providing solutions to food insecurity and hunger provides additional benefits to devoted organizations. Although delivering food to families who have been experiencing low and marginal food security may not produce long-term benefits, the singular solution of supplying food has remained consistent for many food-security initiatives throughout the world [7,17,18]. Such singular commitment to specific solutions may cause organizations to miss opportunities to embrace new innovations in improving food security.

Organizations committed to specific nongovernment food programs also position themselves to influence others in the community [12,13]. For example, if a ministry group acts with dedicated commitment toward a nongovernment food program to hunger, other organizations not originally involved may pay attention and get involved or pioneer their own solution. Commitment to nongovernment food programs can influence involvement and also how the community discusses and thinks about those utilizing their programs [[19], [20], [21], [22]]. This inherently influences whether decisions about how to address food insecurity and hunger are made locally, nationally, or globally, and how to think about those who are using these services.

This study investigated and articulated which aspects of nongovernment food programs engender commitment from community organizations. Four research questions were posed. First, what is the problem these organizations are aiming to address through their nongovernment food programs? Second, how do they identify themselves? Third, what do organizations want from their programs? Fourth, what are their assumptions about nongovernment food programs and their participants? These questions were investigated via qualitative interviews with key actors at a food bank, faith-based organizations, and schools. By answering these questions, we expected to enhance understanding of how and why organizations in local communities form commitment toward alleviating food insecurity and how the basis for their commitment influences the policy and programmatic actions that they take.

## Methods

### Policy framework

Our study was guided by 2 components of Lassell’s policy sciences framework: problem orientation and social process [10]. Problem orientation concerns how individuals and groups view the problems and what goals they have. Social process concerns how “individuals and groups interact, adjust and readjust and establish relationships and pattern of behavior which are again modified through social interactions” [23]. Social process is mapped by examining the perspectives of participating individuals or groups in the process and the base values (i.e., assets or resources) that they use to achieve their goals. Perspectives are understood through examining with whom participants identify, what participants want, and what their assumptions are. Base values are power, knowledge, wealth, well-being, skill, relationships, respect, and rectitude [10].

### Study design

This study collected and analyzed observational and interview data (*n* = 36 participants) on the day-to-day operations of 1 food bank (*n* = 3), 17 faith-based organizations (*n* = 19), and 13 elementary and middle schools (*n* = 14) that shared in the responsibility of providing nongovernment food programs in South Carolina. The programs included provided school backpacks, food pantries, snacks, breakfasts, food boxes for holidays and parents, school stores, lunches for homeless persons, and senior meals. The study protocol was approved by the Institutional Review Board at the University of South Carolina. Data collection was conducted over 5 mo from March to July 2015, and analysis was conducted from August 2015 to April 2016. Faith-based organizations and schools were selected via purposeful maximum variation sampling and snowball techniques. Criteria included variation in the demographics of the organization, religious affiliation, location, size of organization, and types of programs provided by the organization. Data collection and analysis yielded detailed descriptions of each individual organization, which are useful for documenting uniqueness and important themes that cut across cases [24,25]. This sampling design allowed us to capture and describe central themes across organizations that might be representative of the range of experiences within the community [24].

Although the data collection for this study took place 8 y ago, the data and the findings of the study remain pertinent because the organizations involved and their reasons for being involved in food-assistance programs, as well as the role of these programs in people’s lives, have not changed [[26], [27], [28]]. The SARS-CoV-2 pandemic exacerbated food insecurity temporarily [29], and higher participation of food-insecure families in both government and nongovernment food-assistance programs was reported during the pandemic paralleling temporary changes during the great recession of 2008 and years following [27,30,31].

### Data collection

All participants were required to be English-speaking and were over the age of 18 y. No compensation was provided for participation. A semistructured interview guide was developed with open-ended questions to capture participant responses [20]. The development of the interview guide was guided by Lasswell’s policy concepts [10] and allowed for careful probing on emergent concepts and dimensions as needed to gain further insight (see Supplementary Material). The director of the food bank was the initial contact who agreed to participate in an interview. Other staff members (i.e., food pantry and school-based program leaders) participated in an interview. The food bank staff provided contacts with some of the faith-based organizations with which they work, facilitating their acceptance to be involved. Key actors in organizations were identified through brief, informal interviews prior to scheduling a longer interview time with those involved with food programs. Via phone, e-mail, and in-person, all participants were made aware of the study objectives and focus, and how their contributions were important to the study. Following the participant’s verbal agreement to participate in the interview, a convenient time and location were set up (e.g., local coffee shops, libraries, and community organization offices). The number of interviews was determined based on reaching saturation of themes. Interviews lasted for 1 to 2 h, and participants were asked to talk about how they understood hunger in their community, how food-assistance programs addressed issues in their communities, what was important to them about food-assistance programs, and their organization’s role and function in their communities. Questions regarding the respondents’ ideas about the history and background of the program and opinions and visions about solving hunger in the communities along with associated probing were included in the interview guide. Field notes were taken after each interview to describe the context of the interview, observations of organizational facility (if relevant), and reflections by the interviewer on the interview encounter. Informational documents (e.g., pamphlets, flyers) from each organization were sought and collected when available. When consent was not given to audio-record, additional field notes were taken during and immediately after the interview. The data were collected using face-to-face (85%) or telephone (15%) methods.

### Positionality

The interviewer (EMF) was a doctoral student being mentored by some of the coauthors (EAF, CEB, RPS, CS). The interviewer did not discuss personal background with the interviewees and attempted to create a comfortable environment for interviewees. Although the interviewer may have been different than the interviewees in educational training and career choice, their interests overlapped with the interviewer’s interest in community efforts and interests surrounding food in the Columbia, South Carolina area. The interviewer intended to make participants feel comfortable when responding to the questions and disregard any age, sex, or race difference they may have had during the interview session. The interviewer’s desire to learn from the participants and student status helped ease participant’s worries of power and knowledge related to educational or social hierarchy. In scheduling interviews, the interviewer visited the organizations, if possible, to build rapport with the participants by letting them know that the interviewer was excited to learn about their organization’s outlook and influence within the community. The interviewer assured the participants that the interview was for the sole purpose of gaining knowledge on their perspectives as actors in their organization related to nongovernment food programs.

### Analysis

The analysis was guided by the policy framework [9,10], which provided a useful set of constructs and subconstructs for articulating how organizations come to a consensus about or decide on an action and how they will implement that intent (Table 1). Four constructs through which organizations can decide or plan actions are orientation to the problem, orientation to the solution, perspectives, and base values. Perspectives derive from identifications, wants, and assumptions. Identification is a process through which individuals consider themselves as members of an aggregate or group. People make demands about that they want based on their values, and their sentiments and assumptions are often portrayed in symbolic ways. Base values are assets and resources that can be used by organizations to help their organization acquire benefits and can be used to help support identities, wants, and assumptions. They can also be used to attain more or different values.

**TABLE 1:** Constructs and subconstructs guiding understanding of commitment

Constructs and subconstructs	Definition
Orientation to the problem	How organizations think about the problem of hunger in their communities
Orientation to solutions	How organizations think the problem should be addressed in their communities
Perspectives	People’s viewpoints that are required to understand differences and similarities and find common interests
Identity	How organizations see themselves in relation to others in the community
Wants	What organizations require out of providing a nongovernment food program
Assumptions	What organizations believe will happen because of their commitment to nongovernment food programs
Base values	Assets or resources used to attain any of the above categories
Power	Make and carry out decisions
Enlightenment	To have knowledge
Wealth	To have money or its equivalent
Well-being	To have health, physical and psychological
Skill	Special abilities
Affection	To have family, friends, and warm community relationships
Respect	Show and receive deference
Rectitude	Ethical standards

Verbatim transcripts, field notes, and any informational documents available were analyzed with the assistance of computer software for qualitative data (MAXQDA plus 2015, VERBI Software, Berlin, Germany). The analytic steps included: *1*) the development of a preliminary code book of anticipated categories, concepts, and dimensions; *2*) 5 experienced qualitative researchers independently coded 3 interviews; *3*) codes and new concepts were discussed from all independent coding of the data; *4*) new codes (categories, concepts, dimensions) added to preliminary code book; *5*) page-by-page comparisons of the codes and differences in the application or new code development was discussed until a consensus was reached between the student and her primary advisor; *6*) final code book with agreed-upon anticipated categories, concepts, and dimensions; *7*) primary author used this codebook to code all interviews while allowing for additional categories, concepts, and dimensions to emerge; and *8*) codes were compared within categories across differing types of organization in order to group similar meanings. EAF compared data interpretations and secured an agreement of the elements of the coding with other coauthors. This process included the initial identification and anticipation of major categories, concepts, and dimensions of concepts, and later, an understanding of the relationships between new categories, concepts, and dimensions from the data, which in turn furthered an understanding of the content related to the conceptual framework. Findings were emergent within each concept, not across all data. To further ensure the quality of the data, the researcher responsible for coding participated in peer debriefing. Data were triangulated through multiple tools (e.g., interviews, field notes, multiple coders). To ensure data quality within the study, the student conducted peer debriefing, advisor consultations, and triangulation with the coauthors (EAF, CEB, RPS, CS). The student achieved triangulation by integrating information from her memoing journals, observations, and field notes completed after each interview, and the qualitative interviews.

## Results

**FIGURE 1:**
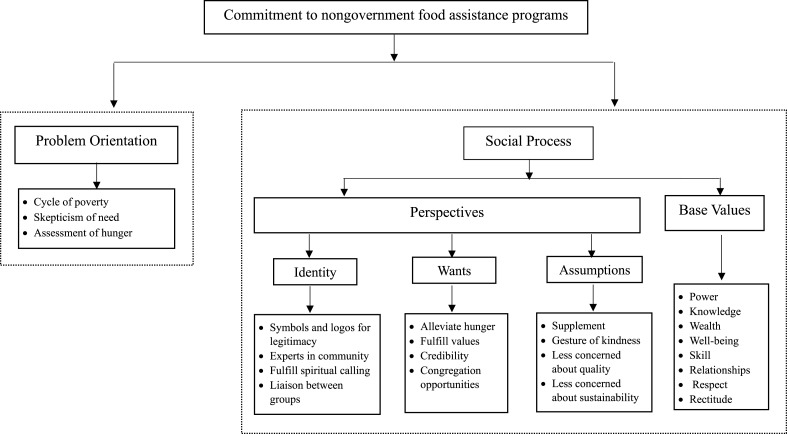
Constructs and subconstructs for understanding commitment to nongovernment food-assistance programs.

A total of 33 interviews with 36 interviewees were completed at 31 different organizations. The diverse sample of participants from 17 faith-based organizations included 11 volunteer leaders and 8 leaders (e.g., pastors). Across 9 elementary schools and 4 middle schools, 12 professional school counselors and 2 principals were interviewed. The director, food pantry leader, and school-based program leader from the local food bank were interviewed for this study. The 33 participants from faith-based organizations, elementary schools, and middle schools had diverse ethnicities or races: 11 participants were White, 7 participants were African American, and 15 participants had mixed racial background. Participants provided insight into how their organizations viewed hunger in the community as the problem, organizational identity**,** what their organizations want from nongovernment food programs, and what organizations assume about nongovernment food programs (Figure 1).

### Orientation in the community to the problem

The interviews unveiled 3 key themes that shed light on orientation to the problem: cycle of poverty, skepticism of need, and how participants assessed hunger (Figure 1).

#### The “cycle of poverty”

Interviewees at food banks, faith-based organizations, and schools understood hunger as an easily noticeable indicator of poverty in their communities. When asked to describe hunger, most referenced hunger as a “cycle of poverty” (Box 1). Although interviewees recognized many families are affected by life circumstances out of their control, they also stated that parents often made poor decisions that negatively affected their families. These choices (e.g., lack of involvement with their children, drug use, and selfishness) were reasons interviewees used to explain why participants in food-assistance programs were reliant on their services. Most interviewees at faith-based organizations also described specific families that were “truly needy” and were sympathetic to their situations (Box 1).

**BOX 1 Quotations regarding hunger in the community as the problem**

***The cycle of poverty***

“Well, a lack of unemployment creates a lack of transportation, inability to afford medicine and medical care…and to have food. They can’t afford their dwelling any longer, can’t afford their utilities, or are disabled. It’s hard to get out of all that.”
“I know that Mom works three jobs, the Dad just up and left her, that has got to be difficult.”

***Skepticism of need***

“We have a few families who work very hard, but then we have most who don’t. They come with their hands out. Every time you turn around, can you help me with this, can you help me with that? I get annoyed because the kids who are on free lunch come with a dollar for snacks – or the backpack kids have on new Nikes. It’s unfair that people take advantage of the programs we have. Our Moms could write a book on how to use the system. They go around and use everything they can to get what they want. Every program I bet. They aren’t truly needy. Really needy kids don’t have on fresh kicks, you know?”
“The child is hungry, and we can feed that one child. I think when you get to the other populations in our community, like parents or the homeless who are adults, it’s hard because they [the congregation] start feeling like ‘why aren’t you working?’ to, ‘why aren’t you taking better care of your family?’ Because you know, you can smell the alcohol on them or see their Mercedes. It comes more difficult to get over that. Kids are just kids. Blameless.”

***Assessment of hunger***

“We just rely on our teachers, rely a lot on our staff, you know, they have their, they put their eyes on their students every single day and so they have a real good gauge of it.”
“We don’t have poor people in this neighborhood, I mean it’s just not an issue in our congregation.”
“Well, we get a lot of stories from the school about Moms who come with their hair done and their kids have nice clothes but they are on food stamps. Well, doesn’t it ever occur to them that maybe if they didn’t get that hair-do that they could buy food instead? I don’t get it. And we don’t get that kind of insight here in our church because we don’t have poor people.”

Alt-text: BOX 1

#### Skepticism of need

Most guidance counselors expressed feelings of skepticism toward families participating in their nongovernment food programs. These interviewees reported they saw families using multiple mechanisms through which they could acquire food (e.g., families enrolled in their nongovernment food programs while also using WIC or SNAP) while also enjoying amenities. These interviewees labeled them as “system abusers” or “not really needy” (Box 1). To help alleviate feelings of skepticism about families participating in food-assistance programs, faith-based organizations purposefully created programs that focused on children. As explained by interviewees from faith-based organizations, children are not to blame for their living or financial situations, rendering it easier for their congregations to support without hesitation or questions (Box 1).

#### Assessment of hunger

The food bank assesses and evaluates community hunger using self-collected statistical data and their own internal surveillance system. Schools reported that they rely on the free and reduced lunch rates to assess hunger, in addition to relying on the observations of teachers and guidance counselors (Box 1). Faith-based organizations reported that most of their information about community needs comes from the food bank and their contacts at schools through which they are providing their food-assistance programs. Most faith-based organizations reported that without this information from these sources, they would not be able to determine the actual needs of the community (Box 1).

The food bank and schools have their own methods for identifying hunger and understanding poverty, whereas most faith-based organizations rely on colloquialisms from the food bank and schools to shape their understanding of hunger. Most faith-based organizations interviewed reported that they believed their congregations were not experiencing poverty. These faith-based organizations reported they relied on information from the food bank and schools to describe poverty to them as they do not regularly see or experience it themselves. Their reliance on this information further contributed to their skepticism about need.

### Organizational identity

During the interviews conducted to explore how participants’ commitments are linked to their organizational identities, the existence of 4 identities emerged: symbols and logos for legitimacy, experts in community, fulfilling spiritual calling, and liaison between groups (Figure 1).

#### Food bank

Interviewees at the food bank described their organizational identity in 2 ways: *1*) a member of the Feeding America food-banking network, and *2*) experts on food in their community. To maintain their identity as members of Feeding America, interviewees reported they assumed roles focused on maintaining ethical standards, providing food to the community, and meeting regulated standards for food distribution (Box 2).

**BOX 2 Quotations regarding organizational identity**

***Food bank: identity and commanding expertise***

“As a member of Feeding America, we have a responsibility to uphold that brand – we need to do things the right way. So, I, as a worker here am responsible to that, too. We need to do this to be part of the national movement to fight hunger. We have to be so conscientious about food safety and distribution.”
“With all of our programs we run, we try to incorporate the logos. It goes on the flyers we pass out to families. We used to put it on the backpacks. We follow the guidelines for some programs so that we can use that logo. It shows that our programs are legitimate and backed by facts.”
“We want to be the experts. We want to be the people you can come to and say ‘hey, how do we do this?’, and we have an answer that’s backed by facts. It’s back by that logo, and everything we do is to fulfill that logo. We do so much more than provide food, we also employ many people, we have the ability to refer people to extra services they need…in return in a way for upholding regulations we get to be this little epicenter for the community.”

***Faith-based organizations: identity, fulfillment, and feeling of good***

“We are Jews, and as Jews and through our synagogue, we must uphold our end and help our neighbors. It’s what is expected from us as individuals, and as affiliates of Conservative Judaism.”
“…and as a Muslim, we are urged to do good. We as individuals must come together and be a group who does well for others. This makes us feel good.”
“I just see it as a, you know, a glimpse of the power of God at work in the world through us. This is our blessing; this is how we can be useful. And um, I thank God for that opportunity.”
“We, as Christians are leaders of good morality. We teach, we help those who need help. We can help our neighbors by providing food, if you can help them or someone else out there, you can help yourself, too by fulfilling what God wants. And what he wants is for us to provide food. Christ said to feed the hungry…we want to do that good.”

***Schools: identity as teachers and caregivers along with opening lines of communication***

“I mean, my opinion about the Moms and all doesn’t really impact what I think should be done and what I can do. It’s like, like I can be skeptical about them but when it comes down to it I am going to give these kids as much as I can regardless about how I personally feel about it.”
“I am a teacher, and that means that while the child is at school, I am responsible for them. If they are worried about food at that moment in time, that is my responsibility. I want for my students to have access to as many programs that may help them as possible. That is my job. If I do that, I can really be what I said I am – a teacher. And I love my students.”
“My job goes beyond 9-5 you know? These kids, they are mine. I feel a sense of responsibility to them. Yes, I am their teacher and I take that seriously. They need me. I can take care of them. I can help their parents understand what they need, sometimes that may be food. I can be that in-between from what they need to getting it and I can do that through programs and getting things from the church for that child.”

Alt-text: BOX 2

Interviewees frequently discussed how the use of symbols, like the Feeding America logos, legitimized their identification and inherent expertise in food distribution (Box 2). In addition to using the Feeding America symbol, food bank interviewees described that providing stable food and logistical assistance to individuals and to other community organizations helped them to be viewed as food experts in their community. Their continued innovative programs created using their specific skills and knowledge about food distribution helped them to convey this to other community organizations (Box 2).

#### Faith-based organizations

Faith-based organization interviewees identified as practicing members of their faiths. All faith-based organization interviewees reported that they were providing a program that fulfilled a need within their community (Box 2).

Most interviewees from faith-based organizations explained that beyond fulfilling a need in their community, their programs must fulfill a spiritual “calling.” Responding to a need in the community that represented their calling provided interviewees with a “feel good” feeling (Box 2). One church pastor explained how he felt “blessed” to know there is a need for food in their community, and that through their community food-assistance program, they can fulfill their obligation to God, the community, and to themselves (Box 2). A similar explanation was offered by another church minister.

#### *Schools*

School interviewees described themselves as teachers and as members of a large safety net to help the children with whom they work and their families. They thought that as teachers, they had a responsibility to provide as many opportunities as possible for their children (Box 2). Recognizing how their students and families experience poverty, interviewees explained that they provided many opportunities for their students to be nurtured and cared for in a school setting, as they may not have opportunities to thrive at home. Most interviewees were able to separate their personal feelings from their professional beliefs. This understanding justified providing food-assistance programs for students, regardless of how interviewees felt personally about the use of the programs (Box 2).

Some teachers identified as being liaisons between students, parents, and community resources. These interviewees explained they feel a responsibility beyond the time their students are in their classrooms (Box 2).

In summary, organizations understood their identities by associating with larger overarching groups they feel are important. Using symbols and acting in ways that are typically associated with those of the larger group, organizations conveyed their identities to the community.

### What organizations want from nongovernment food programs

Four themes emerged for what organizations wanted: alleviate hunger, fulfill values, credibility, and congregation opportunities (Figure 1). Every organization shared a common perspective on hunger, viewing it as a tangible manifestation of poverty that demanded their attention within communities. Ensuring the emotional and physical health of children was of particular interest to organizations. Despite this shared perspective on hunger, there were differences among organizations in their additional wants (Box 3).

**BOX 3 Quotations regarding what organizations want from nongovernment food programs**

***Fulfilling a need***

“I would hate it if I was hungry. Doing this, I can really help someone who needs it. I think we all just want to help out those who are less fortunate than us. I wouldn’t do a program if I didn’t think it was helping.”
“Here with the food bank and other programs – it’s all about health. And I mean, you know, both mental and physical. We want our people who are using the programs to not really be worrying about it and to also be nourishing their bodies the best they can.”

***Food bank: creating a sense of community***

“We want to help the hungry. That’s our first mission. But a close second is really tapping into the interests of the community and gathering support for our anti-hunger campaign. That’s our mission. If we can create programs that you know, um, entrap people into caring about hunger that is a good thing. Then they will care about their neighbor. We are happy to facilitate that. Without donations and community support we wouldn’t be able to feed those that need it. So, it’s a delicate balance, you know? Having to have a program that addresses needs and is something that the average person can get invested in.”

***Food bank: ethics and standards***

“Being part of Feeding America does a lot for us. We get our guidelines from them on how to store food, acquire food, all that stuff. And by following that, I think builds our reputation in the community. Doing those things in that way that is approved by such a big company kind of, proves that we know what we are doing. You know? We also get a ton of information about hunger and nutrition from Feeding America and our own hunger study that we can give to our churches and schools.”
“We don’t have a lot of wiggle room. We follow orders and regulations by what’s right through Feeding America and the food industry in South Carolina. So if that means we can’t work with a particular church to have them have a pantry because they don’t have the proper spaces, then we try to work with them in another way, like a food drive where they would bring food here. It’s not that we don’t want to – it’s that we can’t and still have our affiliations which are more important to our overall goal of being part of Feeding America, and of fighting hunger.”
“I’m not just concerned about those who need, I’ve got a whole bunch of employees here and their families and so forth. So, in a sense, the community needs to support these programs so that we can support ourselves. We need volunteers and interest to survive.”

***Faith-based organizations: congregation engagement and benefits***

“But our goal is to get people in need back up on the ridge, find out what they need and help them to it. But we also need our main goal, if we succeed is having people get here on Sunday, and it’s how we’re helping people have a mindset to invest in the community, invest in friendships, and using their skills and gifts to do that.”
“The programs provide just the experience of caring for our members.”
“It is very important in a ministry that if you are taking their money, their time, that the people feel like they are doing a good thing. They like to get together to see each other every week. The backpack program provides us with the opportunity to satisfy a reach of different types of congregants. If people don’t want to pack, they can pay for it.”

***Faith-based organizations: trusting other community organizations***

“Well, some of their regulations seem a little wonky if you ask me… I don’t have to understand it all, only that it’s the right way of doing it, I guess.”
“We’ve got the brawn – they’ve got the brains. It’s best not to ask questions!”

***Faith-based organizations: independence***

“We have actively and purposefully decided to not work with [food bank]. For us, we’ve decided it’s important that we pick the food, we pick how it gets delivered, and we get to decide how we do things. They have too much say in what we would do for all the money we pay them; we can figure it out ourselves. Our congregants need to feel the responsibility of doing it themselves. I don’t want to deal with their regulations and rules, so I don’t.”
“It took a little while to start up but I think that was to be expected because we kind of had to re-invent the wheel by ourselves. Logistically and timewise working without them is harder. But I’m willing to come in early or leave late on Sundays so that when it comes down to it, I can make decisions for our church without having to run to someone else. It feels good to do it our way.”

***Schools: awareness and communication about poverty and hunger***

“We have a great relationship with our church, and I always try to be honest with them. I tell them ‘hey – half the kids are being dropped off in fancy cars we might want to rethink some things’ to ‘hey, this family ate because of y’all.’ I just want them to know the whole picture so they can make the decisions based on that.”
“You never know what is going to touch an individual. It’s my hope that the church will see the kids and their families and take pity, and want to help them again and again. Maybe with food, maybe with something else, that’s what I want.”

***Schools: putting students first***

“We want the kids to have access to help and resources if they need it. That is what I want out of this program. I think that’s the whole point for us and for me – to give students what they need. If that means that I have to do extra on Fridays, then so be it. I don’t know if it helps and it’s not about that. Access and the hope that it would help is the point.”
“It’s annoying, these programs they push on me to do. But it’s not for me. It’s for the kids and that’s why I do it.”
“I guess my role in all of this is just providing the program. They make the decisions and I do it. That’s part of the job. I feel like a cog in a wheel, but I do what I am told.”
“Oh! I’ve told them. I’ve told them a million times – I’ve told my principal, I’ve told my church lady that I know, and when the [food bank] person came here – oh, I let him know all right about the issues I have with this whole thing. But I don’t want to make waves. So, I keep my mouth shut and do my business. I don’t want to get fired or written up, and I don’t want to jeopardize things for the kids. It’s like, I know what’s going on, the church knows what’s going on, and they still want to do what they want to do.”
“I you know, have mentioned a few times that I think mentoring or another type of program would be better for the kids. Like, they get a meal here sometimes two and a snack. I told the church this and they want to do what they want to do. These kids they need a role model especially the boys I think more than more food, you know. But I mean, my suggestions didn’t work out too well. It’s just kind of what it is. It’s not worth fighting over and losing the whole program.”

Alt-text: BOX 3

#### Food bank

The food bank wanted to ensure that, through their food-assistance programs, they create a sense of affection within the community. Interviewees reported by providing food-assistance programs, their organization gained attention and recognition in the community. The food bank believed that as other organizations begin to realize the overarching role the food bank has in alleviating hunger in the community, they will come to respect their authority, and the food bank will become attractive for volunteers and donations (Box 3).

Wanting to remain credible in the community while fulfilling the requirements set by Feeding America, the food bank had to establish rectitude in how they provide food. By following Feeding America’s strict regulations, they can fulfill ethical standards required to properly house and distribute food while commanding power through their affiliation with Feeding America. They are also able to provide knowledge about hunger to other organizations (Box 3).

Food bank interviewees also revealed they rarely compromised on large issues with any faith-based organizations or school to provide food-assistance programs. They wanted to uphold the strict Feeding America regulations and were unwilling to waiver in any major way. Interviewees explained that compromising could jeopardize their relationship with Feeding America. If faith-based organizations and schools were unwilling to adhere to their requirements, then they did not work with them in that capacity (Box 3). One food-bank interviewee reported, however, that it was important the programs they provide resonate with the community while adhering to Feeding America’s standards. According to this interviewee, the food bank is responsible for the well-being of the community and of its employees (Box 3).

#### Faith-based organizations

Faith-based organizations wanted to provide a meaningful program that supports well-being for participants in food-assistance programs, one that is attractive and interesting to their congregations. Faith-based organizations wanted an active and community-oriented mission to provide charity experience to their congregations, external affection opportunities by providing interaction with outsiders from their congregations, and internal affection opportunities by providing a social opportunity for their members (Box 3).

Some faith-based organizations described that providing these benefits was more important than the program’s mission. Food-assistance programs provide an opportunity for congregants to become enlightened about their community by learning responsibility, compassion, and a greater understanding of the community outside of their organization. They also provide a variety of ways in which members can participate in charity by providing various skills and resources (e.g., they can donate money, help in a pantry, or deliver backpacks) (Box 3).

Faith-based organizations working with the food bank explained they did not have the skills to create and implement nongovernment food programs on their own. Often, these faith-based organizations reported they did not understand the reasons for regulations or requirements put in to place by the food bank to work together, but they were willing to relinquish some of their control over design decision for the programs for the assurance of rectitude, i.e., ethical standards (Box 3).

Faith-based organizations that choose to provide food-assistance programs without the partnership of the food bank realized that acting alone is more difficult and requires extra dedication to details. For these faith-based organizations, the benefits of working alone outweighed any logistical or creative inconveniences (Box 3).

#### Schools

Interviewees at schools reported they wanted to provide enlightenment and insight about poverty and hunger to the faith-based organizations they work with by sharing stories about food program participants. Interviewees believed sharing their experiences would help faith-based organizations make the best decisions when choosing to commit to supporting nongovernment food programs (Box 3).

Creating honest and open communication with faith-based organizations also helped schools establish affection in the community and possibly create future opportunities for faith-based organizations to help their students and families (Box 3).

All school interviewees explained that providing food-assistance programs added responsibilities to their jobs, which compelled them frequently to put aside their personal well-being, knowledge (about program participants and the best way to administer nongovernment food programs), and opinions about their food-assistance programs to provide them to students (Box 3). Some interviewees explained when they did speak up about their concerns, their contacts at their faith-based organizations or food bank met them with apprehension and unwillingness to listen. To foster relationships and for the future of food-assistance programs, most interviewees did not offer suggestions for fear of the programs being pulled from their schools, losing their jobs, or angering the faith-based organizations (Box 3).

Interviewees at schools explained that by providing food-assistance programs, they could provide students and families with as many opportunities for assistance as possible. They wanted to create a sense of community, help students and families, and maintain their identities as teachers. They reported that, to provide food-assistance programs, they were willing to prioritize their students’ well-being in addition to other responsibilities they have at school.

In summary, organizations actively wanted to fulfill values they believe will enhance their organizations or to help fulfill their identities. All organizations wanted to provide a helpful service to their community and understand how their food-assistance programs to do this. To fulfill their identities, organizations made decisions about which values were most important to them that must be fulfilled to achieve this goal, sometimes requiring that they compromised on values that were less important to them. These value-for-value transactions used to achieve what organizations wanted were a powerful way of communicating what was most important in the basis of commitment for each organization.

### What organizations assume about nongovernment food programs

Interviewees’ assumptions were tied with 4 themes across organizations: supplement, gesture of kindness, less concerned about quality, and less concerned about sustainability (Figure 1). They described 3 types of program participants while explaining their assumptions: *1*) those who would starve without food-assistance programs, *2*) those who were using nongovernment food programs as a supplement, and *3*) those participating in food-assistance programs in which the program was meant to be a gesture of kindness. Some interviewees across organizations understood food-assistance programs as urgent and necessary for the survival of program participants (Box 4). Providing families with some food regardless of nutritional quality was worthwhile for most interviewees as they believed providing something is better than nothing. When program participants wanted to accept old, ill-packaged, or expired food, it further proved organization interviewees’ beliefs that without food-assistance programs, some would go hungry.

**BOX 4 Quotations regarding organizations’ assumptions about nongovernment food programs**

***Nongovernment food programs are necessary for survival***

“The families we help would starve without the food boxes and backpack programs we give to them.”
“…something even a candy or fruit snack is better than nothing.”

***Nongovernment food programs as a supplement***

“The pantry is just to help them out when they need it. I mean, we have plenty of food but we also need to serve as many people as we can. I have enough meat here for three months but what good is that if I just give it to one family? We want to spread it out. We could never afford to help 300 families for the entire month.” (A church interviewee)
“No, I believe it’s supposed to be helpful but not everything. I mean that’s ridiculous to think that we could feed an entire family for a week, let alone multiple families.” (A church interviewee)
“It’s not supposed to be a whole meal plan. I mean I know the backpack program says that it’s supposed to feed the child for the entire weekend. But I don’t see that being the reality, it’s mostly snacks or what I would think is snacks. It’s just helping a little bit and I think anything that’s extra is always a good thing.” (A school interviewee)
“Food boxes are supposed to be a big amount to help them through the end of the month, maybe for a few days or so. Or they can spread out that food and make it last – I don’t really care either way. The point is, it’s supposed to be helpful.” (A food bank interviewee)
“We get a lot of food back from Hispanic families because they don’t eat meatballs and stuff. I heard this from [another guidance counselor] too. And we just kind of laughed and I said, ‘well that’s what we have so they should get used to it here!’”
“The food is supposed to help them, right? So, if that’s the case, they can use the food for other people that they have over if they don’t want to eat it themselves.”
“You know if you’re truly hungry, you’ll eat what you get, you know what I mean.”
“We have a nutritionist on staff, so when we get to order food we order good food.”
“We get pre-ordered food on pallets for our backpack and holiday boxes. All that food has been picked by specialists at Feeding America to make sure things are nutritious.”

***Nongovernment food programs as a gesture of kindness***

“Some kids get the backpack and I know they don’t need it. But they do need an excuse to come see me on Fridays so picking up the bag for them is huge. We talk for a little bit, give them a hug, and they get their food. It’s not about them being hungry for those kids.”
“It’s just about showing them that someone out there cares about them. I know that it’s not going to end them being hungry forever, but it’s a nice thing to be able to give for students that have it really hard at home.”
“I’m not delusional, I know the food doesn’t keep them from starving. I think it helps though. I think it helps more that the kids know someone somewhere is thinking about them. Like, ‘this cheese-it bag came from someone thinking about me and my family’. Does that sound crazy? I just think the kids can pick up on, you know, what the real thing behind it is.”
“People know when they receive things that it was packed and made with love. So that’s what we are selling- or, or giving to them, love. And they can feel that so even if it’s not about the food, it is in a way.”
“It’s not about the food.”

Alt-text: BOX 4

Interviewees who believed food-assistance programs should provide participants with food as a supplement denied the notion that their services could sustain a family for a month, or a week, depending on the program (Box 4). Describing food-assistance programs as a supplement allowed interviewees to not worry as much about how much food the program was providing participants, as the object of providing the food-assistance programs was to help participants through a period, not sustain them (Box 4).

For faith-based organizations and schools, framing food assistance as a supplement meant that quality of the food was relatively unimportant. Many interviewees from faith-based organizations explained that they enjoyed putting candy and juice boxes into their prepacked backpacks from the food banks because they reasoned parents could use the money that they would have spent on snacks to buy healthier foods for the entire family (Box 4). Likewise, school interviewees explained that food-assistance programs may not be able to provide the appropriate ethnic foods for families but felt assured families could use the food instead to help their children assimilate into American culture or for entertaining purposes.

Food bank interviewees who understood food-assistance programs as a supplement placed emphasis on providing adequate nutrition through their programs. When able, the food bank tried to provide the most nutritious food to its clients (Box 4). Many food-assistance programs that were part of Feeding America’s initiatives were monitored for nutritional value (Box 4)*.* By the nature of the food bank accepting donations from the food industry and individuals, however, the bank also received food that had little to no nutritional value (e.g., condiments). Often, this food was added to preordered food to bolster the packages of what they were providing to program participants. Defining the role of community food-assistance programs as supplemental allowed for the programs to provide food that otherwise would not be acceptable if the goal of community food-assistance programs was to provide adequate, culturally appropriate, nutritional food items.

School interviewees frequently reported food-assistance programs served as a symbol of kindness, rather than as a supplement to their food supply at home. Often the students came from struggling families, where food was not as important as disruptions at home (e.g., a divorce, child neglect). Food-assistance programs provided a way for interviewees at schools to show that each student was cared for and loved (Box 4). Faith-based organization interviewees expressed this same sentiment, sharing that the goal of their nongovernment food programs was to provide some food along with a lot of caring (Box 4).

In summary, most organizations reported their food-assistance programs served as a supplement to participants’ diets or as a symbolic gesture of caring. Framing food-assistance programs in this way allowed organization interviewees to justify the quantity and quality of food provided to program participants, as their programs were not intended to sustain families in the long term.

## Discussion

Commitment of the interviewees in this study was based on how nongovernment food programs aligned with their organizational identities, what their organization wanted, and what they assumed about their involvement in addressing hunger, all while using base values to underly their commitment. Nongovernment food programs enable organizations to meet a real need in their communities, and commitment to these programs fulfills their identities, wants, and assumptions. Hunger is a tangible symptom of poverty that organizations stated they can address by providing food. Organizations mostly relied on local and national statistics, and sometimes colloquialisms from other organizations form views of participants in nongovernment food programs. Only school interviewees consistently reported they had “hands on” experience with program participants.

Organizations used nongovernment food programs to pursue identities important to them. School interviewees identified as caregivers to students, using the base value of affection. The food bank and faith-based organizations relied on symbols (e.g., logos, name recognition) to express the legitimacy of their nongovernment food programs. Interviewees at faith-based organizations also reported that the organizational identity was directly linked to their religious affiliation. Most faith-based organizations reported that providing food to the poor was an obligation of their faith and doing so helped them to achieve their religious identity. All organizations reported wanting to meet a need in the community and provide a safety net for families experiencing hardships. These findings are consistent with those from a study conducted by Eikenberry [32] where respondents from social welfare organizations identified congregations as key players in social welfare provision through contributions such as collaborative food pantries. Respondents also emphasized concrete contributions to the community as core to congregational activity, such as feeding the poor, addressing physical needs, and providing physical and social support to the community for addressing poverty [32].

Food banks and their related outreach programs give the impression of effectively responding to hunger because of marketing and the sheer amount of effort they require to function successfully [5,17,33,34]. This study supports these findings while highlighting that most organizations assumed that the purpose of their nongovernment food programs was to either serve as a supplement for hungry families or to be a symbolic gesture of good will. These assumptions helped justify the quantity, quality, and frequency in which they provide nongovernment food programs. They understood hunger as part of the larger issue of poverty in their communities but passed judgments on nongovernment food programs’ participants. They also reported that their programs were not helping participants long-term and did not address the root causes of participants’ hunger. Based on these assumptions and understandings about their programs, organizations reacted adversely to participants’ attempting to acquire additional food resources (e.g., using additional nongovernment food programs, government initiatives).

Nongovernment food programs helped organizations fulfill their identities, wants, and assumptions while confirming their orientation to addressing the problem of hunger. School interviewees sometimes explained to their corresponding faith-based organizations that they believed hunger was a lower priority for these children than other needs (i.e., mentoring and role models). The need for mentoring was related to the challenge of poverty but not necessarily to hunger specifically. School interviewees explained that they understood that hunger stems from other issues at home, and mentorship could help students have more positive role models in their lives. When school interviewees suggested this to their faith-based organizations, they were met with resistance, with the explanation that providing food is the only type of program of interest to their congregations. An organization’s resistance to solutions other than giving food was often an attempt to preserve the food-assistance system that was currently satisfying members’ wants, needs, and assumptions. This is consistent with existing research that reported that nongovernment food programs conceptualized as part of national food-banking systems are primarily focused on self-preservation, not advocacy for the poor [5,34]. The commitment from organizations to nongovernment food programs leaves little space for other solutions because the food-assistance system has also heavily influenced their thinking about program participants themselves.

Instead of viewing program participants who used opportunities available in the community (both government-supported and other private programs) to help their families as resourceful and innovative, interviewees categorized them as “system abusers” and “not really needy.” Interviewees’ commitment to this system influenced their assumptions about what their programs should provide (e.g., whether serving as a supplement or as a gesture), and, because of limited interactions with program participants, might also further substantiate their assumptions and skepticism about program participants.

Respondents from different organizations mentioned already having some base values that were employed to possess more values. For example, the food bank used their position with Feeding America to secure relationships in the local community with other organizations (e.g., faith-based organizations and schools), thus using power to acquire affection. Through their position of power, the food bank had the ability to accrue enlightenment, in that information about best practices, national statistics, programming, and local information were readily available to them. The food bank used their power to command most of the resources required for nongovernment food programs (e.g., finances, food, space, volunteers), making themselves an appealing partner for faith-based organizations looking to provide nongovernment food programs. Affection through nongovernment food programs at schools is achieved weekly when church members drop off food to a school pantry, and the guidance counselor informs the members about the needs of the students. Well-being is addressed as nongovernment food programs inherently are focused on providing food to those who are hungry.

Many faith-based organizations reported that providing nongovernment food programs that were based on best practices and backed by a reputable organization (i.e., Feeding America) was worth the stringent requirements to provide the programs. This is an example of how the acquisition of base values in favor of others was reported in our data. Acquisition of values was frequent with our school interviewees. Interviewees reported that they made a conscious decision to give up their power to make decisions about the nongovernment food programs they provide in favor of the values of affection and well-being. Interviewees at schools hoped that this acquiescence of power would allow them to create a healthy, supportive environment for their students and their families.

This study used qualitative methods that provided the opportunity to the interviewees to voice their perspectives and experiences with nongovernment food programs. As part of the sampling design, any stakeholders who had not been part of their organizations’ nongovernment food program for less than 3 y were not interviewed. As the sample was diverse, the common themes presented from all organizations reflected the experiences and commonalities across community organizations. The sample, by design, consisted of 1 to 2 stakeholder interviews per organization, so the study may not have captured the beliefs and opinions of all who were involved with nongovernment food programs at their organizations. South Carolina is predominantly rural and culturally and politically conservative. Consequently, results from this study may not be generalizable to all food-banking systems (particularly the inclusion of faith-based organizations’ relationships with schools) in other regions of the United States. Moreover, because this study was based on the perspectives and experiences of the interviewees, social desirability, although unlikely, could have affected some responses.

Nongovernment programs ostensibly are meant to complement government programs and other actions to alleviate food insecurity, but the commitment to these nongovernment programs is based not just on whether they address the problem of lack of food. Commitment derives from the identities, assumptions, wants, and base values of the participating organizations and the people in them. Seeking to improve the well-being of the community by ending hunger was not the primary value that organizations held; instead, the fulfillment of other values (e.g., forming or maintaining relationships within the community), maintaining identity, and appealing to their participants strengthened organizations’ commitment to nongovernment food programs. Nongovernment food programs are provided through a complex system involving multiple organizations with differing and sometimes conflicting orientations to the problem and perspectives. Although the SARS-CoV-2 pandemic exposed vulnerabilities in food systems and supply chains, prompting reflection on the systemic issues contributing to food insecurity [35,36], such reflection is unlikely to alter nongovernment food programs and organizations’ commitment to providing them because of the bases of that commitment. That the primary basis for commitment to these programs was not alleviating food insecurity means that consideration of the root causes of food insecurity was neglected, and the pursuit of innovative alternatives that could be more effective for families was stifled.

## Author contributions

The authors’ responsibilities were as follows—EMF, EAF: designed research; EMF: conducted research; EMF, EAF analyzed data; EMF, EAF, SS, ALR, CEB, RPS, CJS: wrote the paper; EMF, EAF, SS, ALR: had primary responsibility for final content; and all authors: read and approved the final manuscript.

## Funding

The authors reported no funding received for this study.

## Data availability

Data described in the manuscript, code book, and analytic code will be made available upon reasonable request pending approval from the authors.

## Conflict of interest

The authors declare that they have no known competing financial interests or personal relationships that could have appeared to influence the work reported in this paper.

The authors report no conflicts of interest.
