# Supplementary material for: Understanding Commitment of Local Food Banks, Faith-Based Organizations, and Schools to Provide Nongovernment Food Programs
Source: Curr Dev Nutr. 2023 Sep 25;7(10):102005. doi: 10.1016/j.cdnut.2023.102005 (PMC10590716; doi:10.1016/j.cdnut.2023.102005)
Supplement: Multimedia component 1 [file mmc1.pdf]

Understanding commitment of local food banks, faith-based organizations, and schools to provide non-government food programs. Eliza M. Fishbein.

**Supplementary Material: INTERVIEW GUIDE**

| | |
|---|---|
| **Interviewer:** | **Location Site:** |
| **Participant # (numerical):** | **Date:** |
| **Start Time:** | **End Time:** |

*Hello, thank you for meeting with me today. I would like to learn more about how* [insert organization] *provides assistance for community members through providing food programs. I am going to ask you about your experiences and look forward to learning about your involvement with your community. If you have any questions throughout or need clarification, please let me know.*

*Did you have a look at the consent form I sent you by email (or fax)?*

*I have a copy with me here.*

*As you read in the consent form, you are free to stop the interview at any time and refuse to answer any question you wish.*

*Do you have any questions?*

*Great. Let's both sign here.*

*Are we ready to start?*

[START TIME and START AUDIO RECORDER]

*I would like to start by asking you a little bit about yourself and about your history with* [insert organization].

*How would you describe your title, or role in* [insert organization]?

*How long have you been part of* [insert organization]?

*What do you like most about it?*

*Is it different from other organizations you have been part of? How is it different?*

Understanding commitment of local food banks, faith-based organizations, and schools to provide non-government food programs. Eliza M. Fishbein.

*What programs related to food does your organization provide?*

**ADDITIONAL PROBES FOR ANY QUESTION:** Can you expand on this? Can you give me some examples? Can you tell me anything else?

| Main Questions | Additional Questions | Guiding Probe Questions |
|---|---|---|
| **1.** Can you tell me a little bit about the history of [insert program]? | **a.** Can you describe the program?<br>**b.** Who was/has been involved with [insert program] from the beginning? Who is involved regularly?<br>**c.** What happens on a daily or weekly basis for [insert program] to function? | How did you make the decision to become involved with [insert program]? |
| **2.** What prompted the beginning of [insert program]? | **a.** How did your organization know that there was a problem?<br>**b.** What is the program intended to do?<br>**c.** Who is the program intended to serve? d. Where did the idea for the program come from? | Where do participants live? What is that community like in general? Why do you think participants agree to use [insert program]? |
| **3.** In our community, the food bank provides support to a variety of organizations for food related programs. [Insert organization] does not work with them. Can you tell me about your relationship with the food bank? | **a.** Has [insert organization] ever had a working relationship with the food bank? In what capacity?<br><br>IF YES: What were their responsibilities?<br>What were the positives of collaborating with the food bank?<br>What were the downsides of collaborating with the food bank? | Has it always been this way?<br><br>What has changed? |
| **4.** IF YES TO QUESTION 3: Can you tell me about a time when there may have been a difference in opinions between your organization and the food bank? | **a.** How did you begin to notice this as a concern?<br>**b.** Did others in [insert organization] see this as a concern? If so, how has it been addressed?<br>**c.** Has there been a time where a change has been made because of opinions or suggestions? | Do you feel as though you can voice your opinions or suggestions? |

| | | |
|---|---|---|
| **5.** Is there anything that you find worrisome about [insert program]? | **a.** How did you begin to notice this as a concern?<br>**b.** Do others in [insert organization] see this as a concern? If so, how has it been addressed? | Do you feel as though you can voice your opinions or suggestions? |
| **6.** Concerning [insert program], has [insert organization] ever worked with another community group, church, or individual other than the food bank? | **a.** How did that partnership form?<br>**b.** Can you tell me a little bit about that [insert program]?<br>**c.** How is that relationship similar or different to that with the food bank? | |
| **7.** In the big picture of the community addressing hunger [or how they identify the problem], how does [insert organization] fit into solving the problem? | **a.** How do you feel [insert organization] is contributing to the community?<br>**b.** Are there aspects of [insert program] that benefit the volunteers as well as the participants? | What do you enjoy most about [insert program]?<br><br>How do you see your personal involvement in the community? |
| **8.** What do you envision for the future of [insert program]? | **a.** How do you envision future collaborations with the food bank?<br>**b.** How do you envision future collaborations with any other community groups?<br>**c.** What do you envision for the participants of [insert program]. | What do you think your role will be in the future of [insert program]? |

*Thank you so much for your thoughtful responses. I really enjoyed learning more about* [insert organization], *and hearing about your personal experiences with* [insert program]. *This concludes our interview. I will follow up with you shortly, and if you think of anything else you'd like to share with me please contact me.*
